# Bluetongue virus infection in naïve cattle: Identification of circulating serotypes and associated *Culicoides* biting midge species in Trinidad

**DOI:** 10.1016/j.vetmic.2017.09.008

**Published:** 2017-11

**Authors:** T. Brown-Joseph, C. Batten, L.E. Harrup, L. Frost, J. Flannery, H. Hicks, V. Ramkissoon, R. Ramdeen, C.V. Carrington, C.A.L. Oura

**Affiliations:** a Department of Preclinical Sciences, Faculty of Medical Sciences, The University of the West Indies, St. Augustine, Trinidad and Tobago; b Non-vesicular Reference Laboratory, The Pirbright Institute, Ash Road, Pirbright, GU24 0NF, UK; c Entomology Group, The Pirbright Institute, Ash Road, Pirbright, GU24 0NF, UK; d School of Veterinary Medicine, Faculty of Medical Sciences, The University of the West Indies, St. Augustine, Trinidad and Tobago

## Abstract

To better understand risks associated with trading cattle, it is important to know which serotypes of Bluetongue virus (BTV) are circulating within the exporting and importing country. Hence, this study was conducted to identify the circulating serotypes of BTV in Trinidad. Blood samples were collected monthly from sixty BTV- naïve imported cattle over a six month period after their arrival in the country. Virological (PCR and virus isolation) and serological (ELISA) analyses were carried out on the samples and CDC light traps were placed near the cattle enclosure to trap and identify the species of *Culicoides* biting midges that were present. All of the cattle seroconverted for BTV antibodies within three months of their arrival in the country and real-time reverse transcription PCR (rRT-PCR) detected BTV-RNA in the samples throughout the remainder of the study. The patterns of infection observed in the cattle indicated serial infections with multiple serotypes. A combination of BTV serotype-specific rRT-PCR on the original blood samples and virus isolation followed by serotype-specific rRT-PCR on selected samples, confirmed the presence of BTV serotypes 1, 2, 3, 5, 12 and 17. This is the first report of BTV-2 and BTV-5 in Trinidad. Light-suction traps placed in close proximity to the cattle predominantly trapped Culicoides insignis Lutz 1913 species (96%), with a further six Culicoides species making up the remaining 4% of trapped samples. The circulation of multiple BTV serotypes in Trinidad underlines the need for regular surveillance, which will contribute to the development of risk assessments for trade in livestock.

## Highlights

• High prevalence of bluetongue virus in Trinidad.
• Identification of six bluetongue virus serotypes in Trinidad.
• First isolation of BTV-2 and BTV-5 in Trinidad.
• Risk of emergence of reassortant viruses.

## 1 Introduction

Bluetongue virus (BTV), an Orbivirus in the family Reoviridae, is a double-stranded RNA virus that causes an infectious, non-contagious disease, bluetongue (BT), in susceptible ruminants, with varying degrees of severity dependent on the host species and the BTV serotype/strain (Caporale et al., 2014). Bluetongue virus is transmitted by hematophagous *Culicoides* biting midges (Diptera, Ceratopogonidae), with *Culicoides insignis* Lutz, 1913 and *C. pusillus* Lutz, 1913 currently considered as the main vector species in the Caribbean and Latin America region (Mo et al., 1994).

Although there are few reports of clinical BT outbreaks in the Americas, BTV is endemic throughout the region (Thompson et al., 1992, Mo et al., 1994). The endemic BTV serotypes found in the United States of America (USA) are BTV-10, 11, 13 and 17. While BTV-2 is present in the Southwestern USA (MacLachlan et al., 2013), it is generally only reported as endemic in southern Florida. In addition, BTV-1, 3, 5, 6, 9, 12, 14, 19, 22 and 24 have been isolated in the USA, but are considered non-endemic (APHIS/USDA, 2016). Extensive studies on BTV in the Caribbean and Central/Latin American regions were carried out in the early 1990s (reviewed by Legisa et al., 2014). These studies identified a high prevalence (79%) of BTV antibodies in ovine and bovine livestock from Trinidad and Tobago (TTO) and identified BTV serotypes 1, 3, 4, 6, 8, 12 and 17 to be circulating (Gibbs et al., 1983). BTV-3 was the only serotype isolated from TTO samples during these studies (Thompson et al., 1992). There have been limited studies on BTV in TTO and the Caribbean region since the 1990s.

The objectives of this study were to identify which BTV serotypes are currently circulating in Trinidad and the implications for trade in the region. The study also set out to investigate the kinetics of BTV infection in naïve cattle over a six month period and to identify the vector species of *Culicoides* which may be involved in the transmission of BTV at the study site.

## 2 Materials and methods

### 2.1 Cattle

Sixty BT-naïve dairy cattle (22 Holstein and 38 Jersey breeds) were imported into Trinidad from the USA in June 2013. The cattle tested negative for BT antibodies during quarantine in the USA, prior to shipment to Trinidad. The cattle were bled on their arrival (month 0) and then monthly for six months to December 2013.

### 2.2 BTV serology

Serum samples were tested for BTV-specific antibodies using a BTV group-specific ELISA (LSIVet Ruminant Bluetongue Advanced II Serum ELISA Kit, France) following manufacturers instructions.

### 2.3 RNA extraction and BTV group- and serotype-specific rRT-PCR

Viral RNA was extracted from EDTA whole blood samples at the Non-Vesicular Reference Laboratory (The Pirbright Institute, Surrey, UK) using MagVet Universal Isolation Kits (ThermoFisher Scientific, Paisley, UK) with the KingFisher Flex Purification System (ThermoFisher Scientific, Paisley, UK). The extracted RNA was then tested by a group-specific rRT-PCR (Hofmann et al., 2008). Selected samples from different cattle with low Ct values in the group-specific rRT-PCR were then tested by serotype-specific rRT-PCR for serotypes 1 to 24 (Maan et al., 2012). All rRT-PCR were carried out using an Applied Biosystems 7500 Real-Time PCR System (ThermoFisher Scientific).

### 2.4 Virus isolation

Eighteen samples from different cattle with low Ct values (<30) in the BTV group-specific rRT-PCR assay were selected for viral isolation. In summary, 200μl washed red blood cells (stored in Dulbeccos Phosphate Buffered Saline (DPBS)) were used to inoculate insect KC cells (derived from embryonic C. sonorensis Wirth and Jones 1952 (Wechsler et al., 1991) and incubated at 25C without carbon dioxide (CO_2_) supplementation for seven days. KC cells were harvested on day 7. Virus isolation was confirmed by BTV group-specific rRT-PCR using viral RNA extracted from the cell suspensions.

### 2.5 Culicoides collection and identification

Every two months during the six month study period, six incandescent miniature Centers for Disease Control (CDC) light-suction traps (model 512, John W. Hock, Gainsville, FL, USA) were placed over night in close proximity to the cattle. The traps were placed away from artificial light sources at distances ranging between 0 m from the cattle and 1.5 m above the ground. Collected *Culicoides* were sorted and identified by morphology to a species level using the biological keys of Aitken et al. (1975).

## 3 Results

### 3.1 BTV seroconversion

All 60 animals tested seronegative for BTV antibodies three days after arrival into Trinidad. By month three, all of the cattle had seroconverted (five cattle seroconverted by month one, a further fifteen cattle seroconverted by month two and another twenty-two cattle seroconverted by month three). By month three several of the animals (n=20) were unavailable for blood sampling.

### 3.2 BTV group-specific rRT-PCR

Blood samples collected from the cattle in months one to six were tested for BTV-RNA by group-specific rRT-PCR. By month three, all animals (n=40) tested positive for BTV-RNA. Seven animals tested positive at month one, with an additional 21 and 19 animals testing positive at months two and three, respectively.

**Fig. 1:**
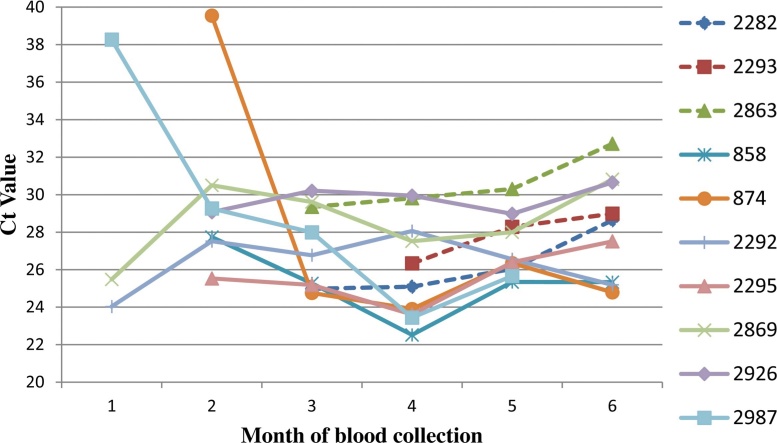
**BTV RNA levels in ten animals over a six-month period.** Whole (EDTA) blood samples from the first month of virus detection to the last month of blood collection (month 6) were assayed by BTV group-specific rRT-PCRs and Ct values were plotted. Dashed lines indicate those samples that demonstrated gradual increases of Ct values with time; solid lines indicate those with sudden declines in Ct values after initial virus detection.

In order to establish the kinetics of infection over the six-month study period, the Ct values from the group-specific rRT-PCRs were plotted for ten selected animals over the six-month period (Fig. 1). Cattle that survived the entire six month period and tested BTV positive by rRT-PCR for at least three consecutive months were selected. Three of the cattle (dashed lines in Fig. 1), displayed a gradual increase in Ct values (indicating a decline in levels of BTV-RNA) following the initial infection. The remaining seven cattle (solid lines in Fig. 1) showed marked decreases in Ct values (indicating increasing viral RNA levels) at various time-points throughout the infection period. Interestingly, for cattle that had been infected for four to five months, the Ct values remained at relatively low levels (Ct values of 24; 28) until the end of the study period.

### 3.3 BTV serotype-specific rRT-PCR

Six samples taken from different cattle with the lowest Ct values (<30) in the group-specific rRT-PCR from months one, two and three were selected (n=18) for subsequent testing using serotype-specific rRT-PCR for BTV serotypes 1 to 24 (Maan et al., 2012). Results indicated the presence of BTV serotypes 3, 5, 12 and 17 (Table 1) and provided evidence of co-infections with two BTV serotypes in four samples (one BTV-5 and BTV-17; three BTV-12 and BTV-17). In five of the samples that were positive in the group-specific rRT-PCR, no serotype could be determined using the serotype-specific rRT-PCR assays for serotypes 1 to 24 (Table 1).

**Table 1:** Detection of BTV-RNA for the first time in 18 animals by group-specific rRT-PCR and the identification of BTV serotypes by serotype-specific rRT-PCR and virus isolation (**-** no serotype detected by rRT-PCR; NSI no serotype isolated).

Month sample taken	Animal Tag ID	Ct values for BTV group-specific PCR assay	Ct values for BTV serotype-specific rRT-PCR assays						BTV serotype isolated
			BTV-1 (n = 0)	BTV-2 (n = 0)	BTV-3 (n = 2)	BTV-5 (n = 6)	BTV-12 (n = 3)	BTV-17 (n = 6)	
1	229	29.99	–	–	–	35.51	–	–	NSI
1	648	25.85	–	–	–	31.00	–	–	BTV-5
1	2292	24.03	–	–	–	–	–	29.89	BTV-17
1	2746	24.35	–	–	–	29.94	–	–	NSI
1	2869	25.48	–	–	–	31.38	–	–	BTV-5
1	No-tags	26.34	–	–	–	–	–	–	NSI
2	847	24.05	–	–	–	–	–	–	NSI
2	857	23.85	–	–	–	34.9	–	33.03	BTV-5
2	858	22.74	–	–	27.4	–	–	–	NSI
2	862	24.40	–	–	–	–	–	29.84	BTV-17
2	863	23.33	–	–	–	–	–	–	BTV-2
2	2287	24.56	–	–	–	–	–	–	BTV-2
3	229	26.58	–	–	–	32.44	–	–	NSI
3	662	25.55	–	–	–	–	33.52	31.29	NSI
3	865	23.41	–	–	33.85	–	–	–	BTV-1
3	2282	24.98	–	–	–	–	–	–	NSI
3	2860	24.15	–	–	–	–	30.31	41.14	BTV-12
3	2888	24.34	–	–	–	–	29.62	30.98	BTV-17

### 3.4 Virus isolation

Virus isolation was attempted on the 18 selected samples above (Table 1). BTV serotypes 1, 2, 5, 12 and 17 were isolated from ten of the samples (Table 1). BTV-1 and 2 were isolated from three samples which did not test positive in the respective serotype-specific rRT-PCR (Table 1). Interestingly, BTV-1 was isolated from a sample 865 which tested positive for BTV-3, but not for BTV-1, by serotype-specific rRT-PCR, indicating that this animal was likely to have been co-infected with BTV-1 and BTV-3. For the remaining three samples (847, 2282 and No-tag), which were positive for BTV-RNA in the group-specific rRT-PCR, it was not possible to identify the BTV serotype by either serotype-specific rRT-PCR or by virus isolation (Table 1).

### 3.5 *Culicoides* identification

A total of 310 *Culicoides* specimens were collected from six light-suction trap catches completed bimonthly during the six month study period, with an average of 103 *Culicoides* collected per trapping session (range: 4–234). Trapped *Culicoides* were predominantly females (n=306) with only four male *Culicoides* collected. The percentages of different *Culicoides* species trapped were as follows: *Culicoides insignis* (94.2%), *Culicoides aitkeni* Wirth & Blanton, 1968 (1.9%), *Culicoides filariferus* Hoffman, 1939/*Culicoides ocumarensis* Ortiz, 1950 (1.9%), *Culicoides pusillus* (1.0%), *Culicoides guyanensis* Floch & Abonnenc, 1942 (0.3%), *Culicoides furens* Poey, 1851 (0.3%), and *Culicoides insinuatus* Ortiz & Leon, 1955 (0.3%).

## 4 Discussion

Trinidad is the southern-most island in the Caribbean, connecting the Caribbean archipelago to the South American mainland, with the north-eastern coast of Venezuela located just 11km south west of Trinidad. Due to its close proximity to South America, Trinidad is highly susceptible to the influx of arboviruses from South America both through the movement of animals and animal products (including illegal movement), and the movement of infected vectors (Sellers et al., 1978, Alba et al., 2004, Burgin et al., 2012). It is known that Culicoides can be passively dispersed in wind currents for up to 100km over water (Kelso and Milne, 2014).

In an attempt to improve the genetic stock of dairy cattle in Trinidad, 22 pure-bred Holstein and 38 Jersey cattle were imported from the USA into Trinidad in June 2013. The arrival of this cohort of BTV naïve cattle provided a unique opportunity to carry out a sentinel study to define the patterns of infection, as well as to identify which BTV serotypes were currently circulating in Trinidad. The cattle were all seronegative for BTV antibodies three days after entry into Trinidad, however all the cattle seroconverted in their first three months in the country. This rapid rate of BTV seroconversion suggests high levels of circulation of BTV in Trinidad.

The predominant *Culicoides* species collected near the cattle under study was *C. insignis*, which is considered the principal BTV vector in the Caribbean (Walton, 2004, Mo et al., 1994, Tanya et al., 1992, Greiner et al., 1989). Its high abundance in an area with active BTV transmission implicates it as a potentially significant vector of BTV in Trinidad. Further work is however required to confirm the role of *C. insignis* in BTV transmission in Trinidad, investigate its role in maintaining transmission of multiple BTV serotypes, and investigate its potential role in facilitating the movement of BTV between South America and Trinidad. Transmission of BTV by other *Culicoides* species, particularly *C. pusillus*, may also be possible (Mo et al., 1994) and further work is required to investigate the relative abundance and distribution of Culicoides species in Trinidad, and their potential role in BTV transmission.

One of the species identified in this study was described by Aitken et al. (1975) as *Culicoides filariferus* Hoffman, 1939/*Culicoides ocumarensis* Ortiz, 1950. This species belongs to the Guttatus species group/complex, which is considered difficult to differentiate morphologically. Molecular methods are therefore required to accurately identify and differentiate members of this group/complex (Harrup et al., 2015).

The patterns of BTV infection (levels of viral RNA) observed during this study were consistent with the cattle being serially infected with multiple serotypes of BTV, as BTV-RNA levels in the cattle increased at various time points over the infection period. Additionally viral RNA levels persisted at relatively high levels (Ct values <28) in many of the cattle 4–5 months after initial infection. If the cattle had been infected with a single serotype, viral RNA levels would have been expected to reduce steadily over the infection period and drop to low or undetectable levels by four to five months post-infection (MacLachlan et al., 1994, Katz et al., 1994, Bonneau et al., 2002, Di Gialleonardo et al., 2011). This observation is supported by confirmation of the circulation of six distinct serotypes ofBTV in the cattle as well as the co-infection of some of the cattle with two BTV serotypes. Further work is required to investigate the prevalence of BTV co-infections among Trinidadian livestock and its implication for onward transmission to *Culicoides* vectors and the potential for reassortment between co-circulating BTV serotypes.

BTV serotype-specific rRT-PCR confirmed the presence of serotypes 3, 5, 12 and 17 in the cattle. However, some samples, while testing positive for BTV in the group-specific rRT-PCR, tested negative by the serotype-specific rRT-PCRs for serotypes 1–24. This suggests that either novel serotypes of BTV are circulating in Trinidad or that variants of serotypes 1 to 24 are present that are not being detected by serotype-specific rRT-PCR. The latter is more likely, as the primers for the serotype-specific rRT-PCR assays were designed to detect BTV serotypes mostly from Europe, Asia and Africa. Further support for this explanation is the large discrepancy in Ct values (in most cases greater than six Cts) between the respective group-specific and serotype-specific rRT-PCR results and the fact that serotypes 1 and 2 (which were not detected by rRT-PCR) were isolated in cell culture (Table 1). Low sensitivity to certain local Caribbean strains due to primer/probe mismatching could also result in some co-infections being missed. The results from animal 865 (which was serotype-specific rRT-PCR positive for BTV-3, but only yielded BTV-1 by virus isolation), indicate that at least one co-infection was missed by the serotype-specific rRT-PCR assay. Sequencing of the VP2 genes of the Trinidad BTV isolates would permit development of better matched primers and probes and would enable further optimization of the serotype-specific rRT-PCR assays for use to identify BTV serotypes from the Americas. The reason why BTV-3 was not isolated from animal 865 is unclear, but it is possible that this particular Caribbean strain of BTV-3 does not grow well on KC cells.

This is the first report of BTV-2 and BTV-5 circulating in TTO, as these serotypes were not detected in studies carried out in the Caribbean around 20–25 years ago (Greiner et al., 1989, Thompson et al., 1992, Mo et al., 1994, Legisa et al., 2014). BTV-2 is however considered to be endemic in southeastern USA, particularly in Florida (APHIS/USDA, 2016), while BTV-5 has been isolated in cattle in the southeastern USA in 1998 (Johnson, 2011), and has also been isolated in Guadeloupe (Legisa et al., 2014). Further VP2 sequencing is required in order to investigate the phylogenetic relationship between the BTV-2 and BTV-5 strains circulating in the USA, Guadeloupe and Trinidad.

Confirmation of the co-circulation of multiple serotypes and the co-infection of different BTV serotypes within cattle in this study supports the likelihood of reassortment of BTV strains circulating in Trinidad. This may lead to the emergence of novel viruses with different phenotypes related to transmission and virulence (Nomikou et al., 2015).

## 5 Conclusion

This study has confirmed the presence and co-circulation of BTV serotypes 1, 2, 3, 5, 12 and 17 in Trinidad, with BTV-2 and BTV-5 being reported for the first time in the country. Culicoides insignis, a known vector for BTV in the Caribbean, was identified to be present along with six other *Culicoides* species. Multiple co-circulating serotypes and strains of BTV present a high risk of genetic reassortment resulting in the possible evolution of novel BTV strains. These results emphasise the importance of carrying out regular surveillance for BTV in order to better inform risk assessments and enable the safe trade of animals both regionally and globally.

## Conflicts of interest

The authors know of no financial or personal conflicts of interest with any person or organization that could inappropriately influence this work. Founders had no role in study design nor the collection, analysis and interpretation of data.
